# Comparing the safety and efficacy of sleeve gastrectomy versus Roux-en-Y gastric bypass in elderly (>60 years) with severe obesity: an umbrella systematic review and meta-analysis

**DOI:** 10.1097/JS9.0000000000000629

**Published:** 2023-10-04

**Authors:** Mohammad Kermansaravi, Antonio Vitiello, Rohollah Valizadeh, Shahab Shahabi Shahmiri, Mario Musella

**Affiliations:** a Department of Surgery ,Minimally Invasive Surgery Research Center, Division of Minimally Invasive and Bariatric Surgery, Rasool-e Akram Hospital, School of Medicine, Iran University of Medical Sciences; b Center of Excellence of European Branch of International Federation for Surgery of Obesity, Hazrat_e Rasool Hospital, Tehran; c Urmia University of Medical Sciences, Urmia, Iran; d Advanced Biomedical Sciences Department, “Federico II” University, Napoli, Italy

**Keywords:** bariatric surgery, obesity, older adults, Roux-en-Y Gastric Bypass, sleeve gastrectomy, umbrella review

## Abstract

**Background:**

Today, bariatric surgeons face the challenge of treating older adults with class III obesity. The indications and outcomes of Roux-en-Y gastric bypass (RYGB) versus sleeve gastrectomy (SG) also constitute a controversy.

**Methods:**

PubMed, Web of Science and Scopus were searched to retrieve systematic reviews/meta-analyses published by 1 March 2022. The selected articles were qualitatively evaluated using A Measurement Tool to Assess systematic Reviews (AMSTAR).

**Results:**

An umbrella review included six meta-analyses retrieved from the literature. The risk of early-emerging and late-emerging complications decreased by 55% and 41% in the patients underwent SG than in those receiving RYGB, respectively. The chance of the remission of hypertension and obstructive sleep apnoea, respectively increased by 43% and 6%, but type-2 diabetes mellitus decreased by 4% in the patients underwent RYGB than in those receiving SG. RYGB also increased excess weight loss by 15.23% in the patients underwent RYGB than in those receiving SG.

**Conclusion:**

Lower levels of mortality and early-emerging and late-emerging complications were observed in the older adults undergoing SG than in those receiving RYGB, which was, however, more efficient in term of weight loss outcomes and recurrence of obesity-related diseases

## Introduction

Highlights

- Complications were more in the older adults undergoing sleeve gastrectomy than Roux-en-Y gastric bypass (RYGB).
- RYGB was more efficient in term of weight loss outcomes in older adults.
- RYGB reduced the risk of remission of type-2 diabetes mellitus.

The social burden of severe obesity in older adults turns heavier^1–3^ with the globally-growing prevalence of obesity with age^4–7^.

Aging coupled with lack of exercise is associated with overall poor health through gradually lowering muscle proteins, increasing visceral fat and resistance to insulin, and causing atherosclerosis, nutritional deficiency, cognitive decline and frailty. Bariatric and metabolic surgeries (BMS) appear the most promising solution to the comorbidities inflicted upon different age groups, especially geriatric populations with class III obesity^8^.

Over the past decade, the frequency of BMS in older adults has increased by three-fold to 10% of the total bariatric procedures performed annually^9,10^. Bariatric surgeons face the challenge of treating class III obesity, especially in older adults^11,12^.

Although sleeve gastrectomy (SG) is the most common BMS around the world^13^, there is no consensus on SG, as the first choice in elderly patients with severe obesity.

Both Roux-en-Y gastric bypass (RYGB) and SG with low risk for complications can decrease weight, treat obesity-associated disorders and improve quality of life^14^, even in older adults^15^. Nevertheless, differences between SG and RYGB in terms of middle- and long-term postoperative outcomes have yet to be elucidated in this age group^16^. There are some concerns about BMS in this population and the outcomes are still debated^17^ and there is a lack of studies due to issues in this age group compared to the younger adults.

Research suggests a higher mortality caused by BMS and fewer benefits such as weight loss in older adults compared to the young^18–20^. Systematic reviews of pooled analyses, however, reported low rates of mortality and complications and successful weight loss outcomes in patients older than 60–65 years^21,22^. Given the concern over the indications and outcomes of RYGB in older adults with class III obesity, comparative analyses are required to be conducted using other procedures in different age groups^23,24^.

The meta-analyses so far conducted to compare RYGB with SG^8,25,26^ have failed to determine definitive guidelines and optimal procedures for comparing the two methods in terms of safety and efficacy in older adults. The present umbrella review and meta-analysis was conducted to systematically evaluate the context and quality of relevant articles and compare SG with RYGB in terms of safety, weight loss and obesity-associated disorders in aged patients requiring bariatric surgery.

## Materials and methods

This umbrella review was performed according to the guidelines stipulated by the Joanna Briggs Institute^27^. This work has been reported in line with Preferred Reporting Items for Systematic Reviews and Meta-Analyses (PRISMA) and Assessing the methodological quality of systematic reviews (AMSTAR) Guidelines^28^. The study protocol was registered at the Prospective Register of Systematic Reviews (PROSPERO). In addition, this review was registered in Research Registry UIN.

### Search strategy

**Figure 1:**
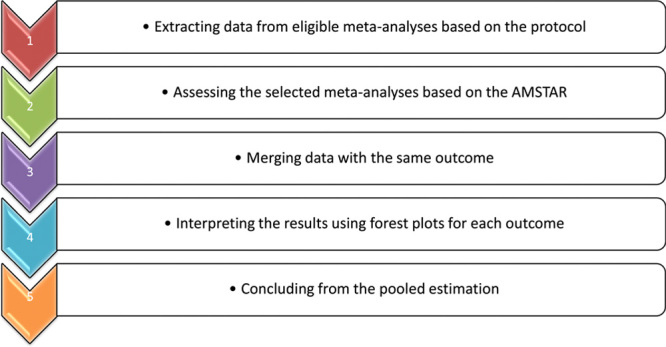
Flowchart of the umberella review.

The keywords used to search in the titles and abstracts of the systematic reviews/meta-analyses published by 1 March 2022 in PubMed, Web of Science and Scopus comprised “severe obesity”, “obesity surgery”, “sleeve gastrectomy”, “bariatric surgery”, “Roux-en Y Gastric Bypass”, “older adults”, “meta-analysis” and their combination. The bibliography of all the selected articles was hand searched to find the eligible articles. After removing duplicate articles, qualitatively evaluating the remaining ones by two of the reviewers yielded an AMSTAR score of at least 10^29^ (Table 1). Figure 1 shows the flowchart of the umberella review.

**Table 1:** AMSTAR score for included meta-analyses.

	AMSTAR Items													
Authors	Uses elements of PICO	A priori research design	Explained selection of the study designs	Comprehensive literature search	Study selection in duplicate	Dual data extraction	Excluded study list provided	Included studies described	Funding sources reported	Quality assessed	Quality used appropriately	Satisfactory discussion of heterogeneity	Conflicts of interest reported	AMSTAR score
Giordano *et al*.^31^	Yes	Yes	Yes	Yes	Yes	No	Yes	Yes	Yes	Yes	Yes	Yes	Yes	12
Giordano *et al*.^32^	Yes	No	Yes	Yes	No	Yes	Yes	Yes	Yes	Yes	Yes	Yes	No	10
Marczuk *et al*.^26^	Yes	No	Yes	Yes	Yes	Yes	Yes	Yes	Yes	Yes	Yes	Yes	Yes	12
Shenoy *et al*.^33^	Yes	No	Yes	Yes	Yes	No	Yes	Yes	No	Yes	Yes	Yes	Yes	10
Chenxin Xu *et al*.^12^	Yes	No	Yes	Yes	Yes	Yes	Yes	Yes	Yes	Yes	Yes	Yes	Yes	10
Vallois *et al*.^34^	Yes	No	Yes	Yes	Yes	Yes	Yes	Yes	No	Yes	Yes	Yes	Yes	11

PICO, Population, intervention, comparison, outcome.

### Statistical analysis

The pooled odds ratios (ORs) of the outcomes of bariatric surgeries, that is RYGB and SG, were considered the main measure of the effect/effect size. Cochrane’s Q test and I^2^ used to compare the articles respectively showed a significant heterogeneity in the meta-analyses and heterogeneity of 0–100%. The random-effects model was used to estimate the dichotomous outcome and pooled ORs as the main index at a 95% CI. The mean difference (MD) was also used to obtain excess weight loss (EWL) based on the DerSimonian-Laird approach. A forest plot was employed to present the pooled ORs of the outcomes of RYGB and SG and Begg’s test to assess publication bias. The number of articles being below 10 made meta-regression and funnel plots inapplicable to publication bias assessments^30^. The data were analyzed in Stats13 by reporting only the mean quantitative variables. The umbrella review was performed through weighting each study by their sample size.

### Data extraction

The data independently extracted from the articles by two reviewers included author names, year of publication, number of primary studies, sample size, early complications, late morbidity, total complications, mortality, early readmission, reoperation, total weight loss, EWL, obstructive sleep apnoea, hypertension and type-2 diabetes mellitus (T2DM). A third investigator independently resolved potential conflicts between the findings of the two reviewers (Tables 2, 3).

**Table 2:** Characteristics of the included studies included in umbrella meta-analysis.

First Author and subject		Year	No. primary studies	Sample size	Perioperative morbidity (early complications) (95% CI)	Late morbidity (95% CI)	Total complication (95% confidence interval)	Mortality (95% CI)	early readmission (95% CI)	Reoperation (95% CI)	%TWL (95% CI)	%EWL (95% CI)	OSA (95% CI)	HTN (95% CI)	T2DM(95% CI)	DLP (95% CI)
Shenoy (SG vs. RYGB on young vs. elderly)^33^		2020	9	2240	0.71 [0.44, 1.13]	0.35 [0.19, 0.65]	—	0.50 [0.15–1.70]	—	—	—	7.79 [−23.96, 8.38]	1.14 [0.55, 2.34]	0.57 [0.35, 0.93]	1.02 [0.63, 1.66]	—
Chenxin Xu(RYGB vs. SG on Young vs. Elderly)^12^		2020	19	31941	1.75 [1.51, 2.04]	1.63 [1.41, 1.88]	—	early: 2.23 [1.37–3.64],Late:1.22 [0.18, 8.06]	1.75 [1.48–2.06]	2.16 [1.67–2.81]	2 year 6.26 [−1.33, 13.85], 3 year 4.97 [−2.34, 12.27]	1 year 19.55 [14.65, 24.46], 2 year 16.56 [0.05, 33.08]	1.31 [0.60, 2.81]	1.73 [1.02, 2.93]	0.89 [0.37–2.13]	—
Giordano (SG on elderly vs. younger)^32^		2020	11	2259	—	—	1.71 [0.76, 3.83]	—	—	—	—	is −7.63 [−13.19, −2.08]	0.81 [0.69, 0.95]	—	—	—
Giordano (RYGB on elderly vs. younger)^31^		2018	7	3128	—	—	1.51 [1.07–2.11]	6.12 [1.08–34.43]	—	—	—	is -3.44 [−5.20 to 1.68] i2:0, p:0.77	—	1 year 0.57 [0.42, 0.76]	—	1 year 0.61 [0.45, 0.83]
Marczuk (RYGB on elderly vs. younger)^26^		2019	9	4391	—	—	1.88 [1.07, 3.30]	4.38 [1.25, 15.31]	—	—		is -5.86 [−9.15, −2.56]	—	0.33 [0.14–0.74]	0.64 [0.42, 0.97]	—
Vallois^34^	RYGB on elderly vs. younger	2020	11	6638	—	—	1.70 [0.98, 2.94]	—	—	—	—	1 year is −5.28 [−7.49, −3.07]	0.97 [0.58, 1.65]	0.42 [0.06, 2.89]	0.51 [0.30, 0.87]	—
	SG on elderly vs. younger	2020	9	26,118	—	—	1.49 [1.28, 1.75]	—	—	—	—	1 year is −4.49 [−6.98, −2.01]	3.36 [0.58, 19.43]	1.13 [0.74, 1.74]	1.69 [0.79, 3.61]	1.37 [0.85, 2.19]

EWL, excess weight loss; HTN, hypertension; SG, sleeve gastrectomy; RYGB, Roux-en-Y gastric bypass; T2DM, type-2 diabetes mellitus.

TWL, total weight loss; DLP, dyslipidemia; OSA, Obstructive sleep apnea.

**Table 3:** Tabular representation of the reported outcomes of the meta-analyses.

Outcome	Meta-analysis	Studies	Patients	ES	95% lower CI	95% upper CI	I^2^, %	*P*
Early complications	Shenoy *et al*.^33^	9	2240	0.71	0.44	1.13	38	0.012
	Chenxin Xu *et al*.^12^	19	31 941	1.75	1.51	2.04	0	0.69
Late complication	Shenoy *et al*.^33^	9	2240	0.35	0.19	0.65	0	0.46
	Chenxin Xu *et al*.^12^	19	31 941	1.63	1.41	1.88	0	0.48
Total complication	Vallois *et al*.^34^ for RYGB	11	6638	1.70	0.98	2.94	76	0.001
	Vallois *et al*.^34^ for SG	9	26 118	1.49	1.28	1.75	48	0.04
	Giordano *et al*.^32^	11	2259	1.71	0.76	3.83	83	<0.001
	Giordano *et al*.^31^	7	3128	1.51	1.07	2.11	0	0.99
	Marczuk *et al*.^26^	9	4391	1.88	1.07	3.30	50	0.05
Mortality	Shenoy *et al*.^33^	9	2240	0.50	0.15	1.70	0	0.45
	Chenxin Xu *et al*.^12^	19	31 941	Early: 2.23; late: 1.22	Early: 1.37; late: 0.18	Early: 3.64; late: 8.06	Early: 37; late: 56	Early: 0.19; late: 0.10
	Giordano *et al*.^31^	7	3128	6.12	1.08	34.43	44	0.13
	Marczuk *et al*.^26^	9	4391	4.38	1.25	15.31	18	0.3
Early readmission	Chenxin Xu *et al*.^12^	19	31 941	1.75	1.48	2.06	0	0.53
Reoperation	Chenxin Xu *et al*.^12^	19	31 941	2.16	1.67	2.81	12	0.34
%TWL	Chenxin Xu *et al*.^12^	19	31 941	2 years: 6.26; 3 years: 4.97	2 years: −1.33; 3 years: 13.85	2 years: 2.34; 3 years: 12.27	2 years: 80; 3 years: 95	2 years: 0.03; 3 years: <0.001
%EWL	Shenoy *et al*.^33^	9	2240	7.79	−23.96	8.38	90	<0.001
	Chenxin Xu *et al*.^12^	19	31 941	1 year 19.55; 2 years: 16.56	1 year 14.65; 2 years: 0.05	1 year 24.46; 2 years: 33.08	Early: 28; late: 80	Early: 0.24; late: 0.02
	Giordano *et al*.^32^	11	2259	−7.63	−13.19	−2.08	84	<0.001
	Giordano *et al*.^31^	7	3128	−3.44	−5.20	−1.68	0	0.87
	Marczuk *et al*.^26^	9	4391	−5.86	−9.15	−2.56	0	0.77
	Vallois *et al*.^34^ for RYGB	11	6638	−5.28	−7.49	−3.07	0	0.71
	Vallois *et al*.^34^ for SG	9	26 118	−4.49	−6.98	−2.01	0	0.74
DLP	Giordano *et al*.^31^	7	3128	0.61	0.45	0.83	0	0.54
	Vallois *et al*.^34^ for SG	9	26 118	1.37	0.85	2.19	46	0.15
T2DM	Shenoy *et al*.^33^	9	2240	1.02	0.63	1.66	0	0.66
	Chenxin Xu *et al*.^12^	19	31 941	0.89	0.37	2.13	57	0.10
	Marczuk *et al*.^26^	9	4391	0.64	0.42	0.97	0	0.54
	Vallois *et al*.^34^ for RYGB	11	6638	0.51	0.30	0.87	—	—
	Vallois *et al*.^34^ for SG	9	26,118	1.69	0.79	3.61	82	<0.001
HTN	Shenoy *et al*.^33^	9	2240	0.57	0.35	0.93	0	0.58
	Chenxin Xu *et al*.^12^	19	31 941	1.73	1.02	2.93	0	0.38
	Giordano *et al*.^31^	7	3128	0.57	0.42	0.76	42	0.14
	Marczuk *et al*.^26^	9	4391	0.33	0.14	0.74	61	0.08
	Vallois *et al*.^34^ for RYGB	11	6638	0.42	0.06	2.89	—	—
	Vallois *et al*.^34^ for SG	9	26 118	1.13	0.74	1.74	39	0.16
OSA	Shenoy *et al*.^33^	9	2240	1.14	0.55	2.34	0	0.70
	Chenxin Xu *et al*.^12^	19	31 941	1.31	0.60	2.81	0	0.56
	Giordano *et al*.^32^	11	2259	0.81	0.69	0.95	0	0.96
	Vallois *et al*.^34^ for RYGB	11	6638	0.97	0.58	1.65	91	<0.001
	Vallois *et al*.^34^ for SG	9	26 118	3.36	0.58	19.43	—	—

EWL, excess weight loss; HTN, hypertension; SG, sleeve gastrectomy; RYGB, Roux-en-Y gastric bypass; T2DM, type-2 diabetes mellitus.

TWL, total weight loss; DLP, dyslipidemia; OSA, Obstructive sleep apnea.

## Results

**Figure 2:**
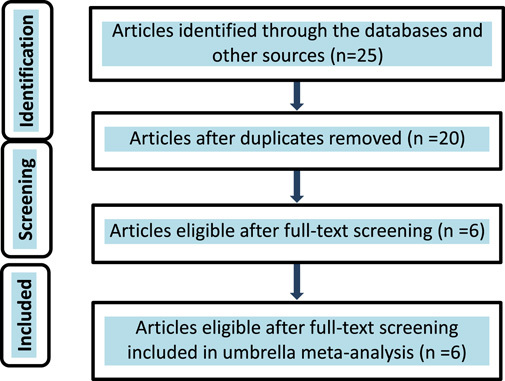
Preferred Reporting Items for Systematic Reviews and Meta-Analyses.

Twenty records were found from our literature search. Of these, 14 were excluded after full-text screening. Totally, we selected six meta-analyses to run umbrella meta-analysis (Fig. 2).

### Early complications for SG vs. RYGB in elderly

**Figure 3:**
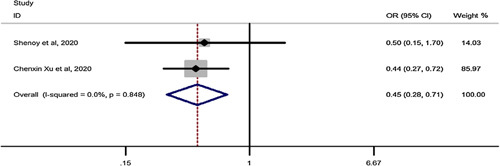
Forest plot to show risk of early complications for sleeve gastrectomy vs. Roux-en-Y gastric bypass in elderly. OR, odds ratio; OSA, Obstructive sleep apnea.

Pooled estimation of a meta-analysis of odds ratio studies reported an OR of 0.45, that is in patients undergoing SG, the chance of early complications decrease by 55% (OR: 0.45, CI 95%: 0.28–0.71) compared to RYGB (Fig. 3).

### Late complications for SG vs. RYGB in elderly

**Figure 4:**
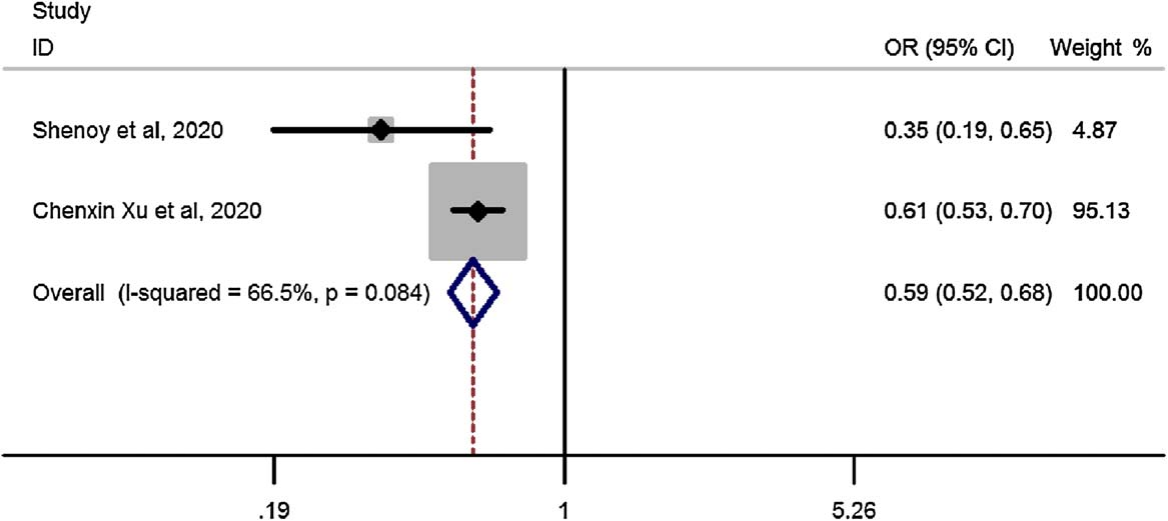
Forest plot to show risk of late complications for sleeve gastrectomy vs. Roux-en-Y gastric bypass in elderly. OR, odds ratio.

Pooled estimation of a meta-analysis of odds ratio studies reported an OR of 0.59, meaning that in patients undergoing SG, the risk of late complications decreases by 41% (OR: 0.59, CI 95%: 0.52–0.68) compared to RYGB (Fig. 4).

### Mortality for SG vs. RYGB in elderly

**Figure 5:**
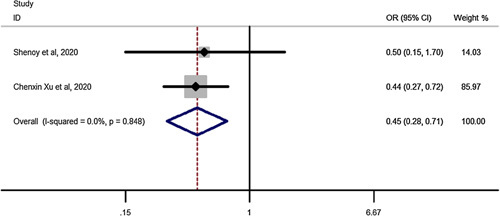
Forest plot to show risk of mortality for sleeve gastrectomy vs. Roux-en-Y gastric bypass in elderly. OR, odds ratio.

Pooled estimation of a meta-analysis of odds ratio studies reported an OR of 0.45, that is in patients undergoing SG, the chance of mortality decrease by 55% (OR: 0.45, CI 95%: 0.28–0.71) compared to RYGB (Fig. 5).

### OSA remission after SG vs. RYGB in elderly

**Figure 6:**
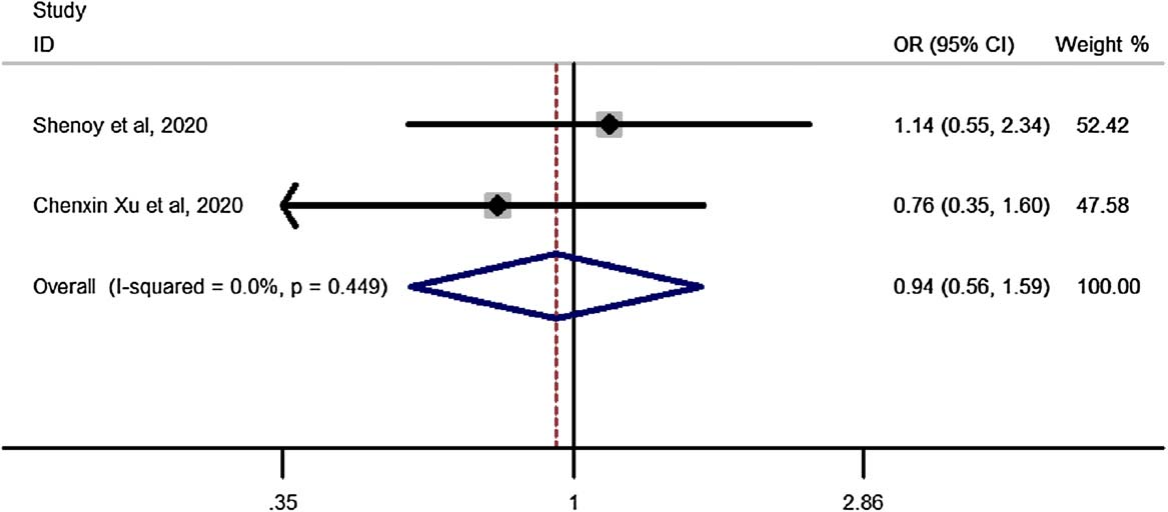
Forest plot to show chance of OSA remission for sleeve gastrectomy vs. Roux-en-Y gastric bypass in elderly. OR, odds ratio.

Pooled estimation of a meta-analysis of odds ratio studies reported an OR of 0.94, that is in patients undergoing SG, the chance of Obstructive sleep apnea (OSA) remission decreases by 6% (OR: 0.94, CI 95%: 0.56–1.59) compared to RYGB but it was not significant showing no difference between SG and RYGB on OSA remission (Fig. 6).

### Hypertension (HTN) remission after SG vs. RYGB in elderly

**Figure 7:**
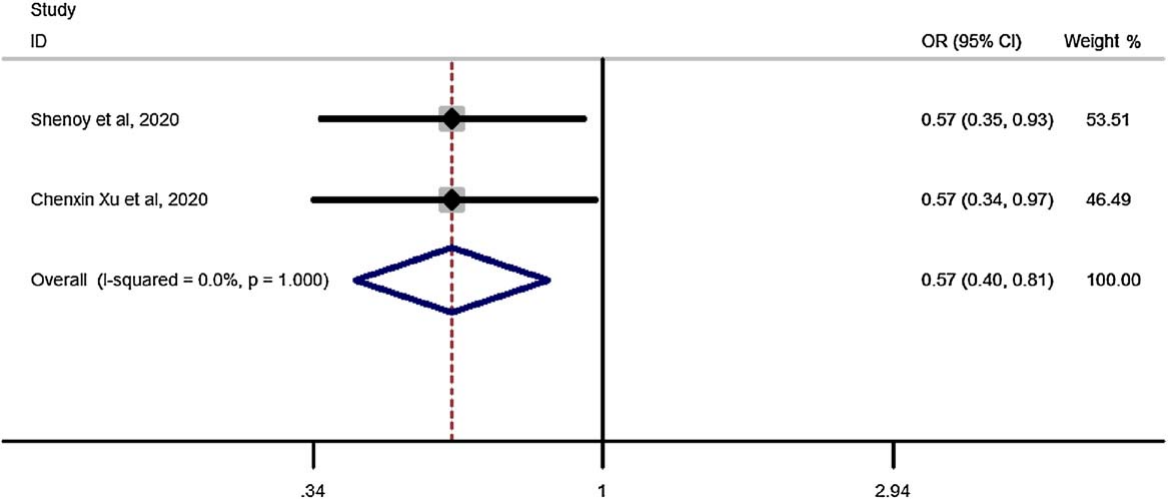
Forest plot to show chance of hypertension remission for sleeve gastrectomy vs. Roux-en-Y gastric bypass in elderly. OR, odds ratio.

Pooled estimation of a meta-analysis of odds ratio studies reported an OR of 0.57, that is in patients undergoing SG, the chance of HTN remission decreases by 43% (OR: 0.57, CI 95%: 0.40–0.81) compared to RYGB (Fig. 7).

### T2DM remission after SG vs. RYGB in elderly

**Figure 8:**
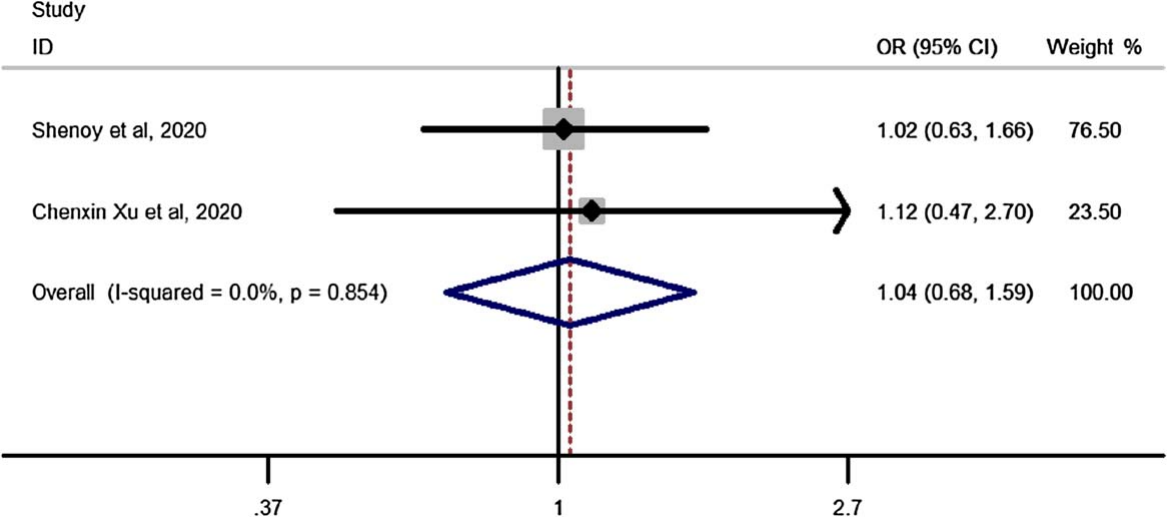
Forest plot to show chance of type-2 diabetes mellitus remission after sleeve gastrectomy vs. Roux-en-Y gastric bypass in elderly. OR, odds ratio.

Pooled estimation of a meta-analysis of odds ratio studies reported an OR of 1.04, that is in patients undergoing SG the chance of T2DM remission increases by 4% (OR: 1.04, CI 95%: 0.68–1.59) compared to RYGB but was not significant (Fig. 8).

### Total complications after RYGB in elderly compared to youngers than 60 years

**Figure 9:**
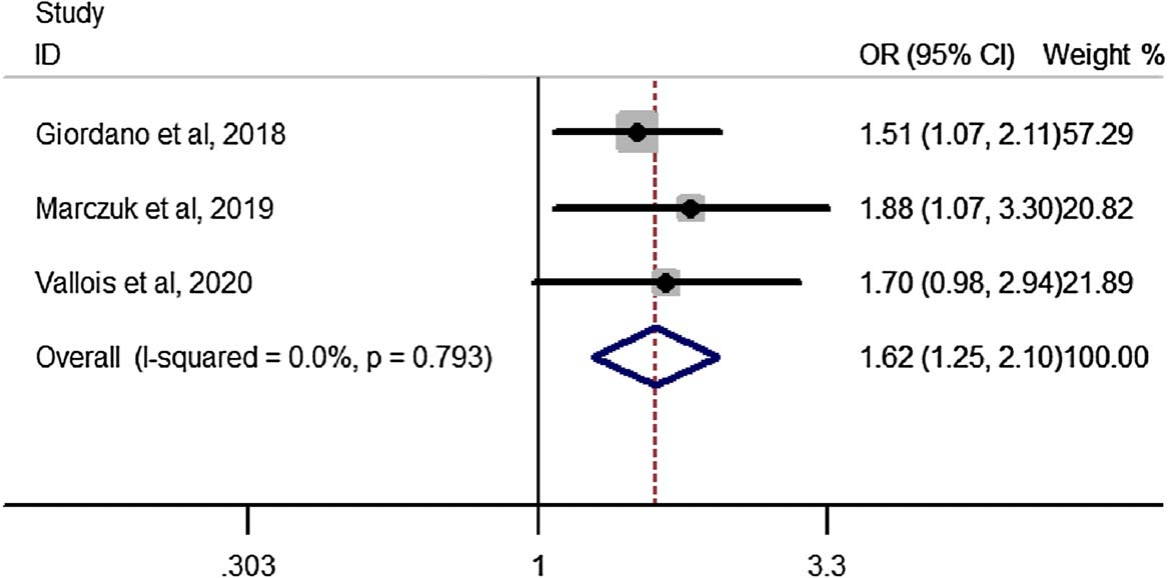
Forest plot to show total complications after Roux-en-Y gastric bypass in elderly compared to youngers than 60 years. OR, odds ratio.

Pooled estimation of a meta-analysis of odds ratio studies reported an OR of 1.62, that is in patients undergoing RYGB bariatric surgery, the chance of total complication increase by 62% (OR: 1.62, CI 95%: 1.25–2.10) in elderly compared to youngers than 60 years(Fig. 9).

### HTN remission after RYGB in elderly compared to youngers than 60 years

**Figure 10:**
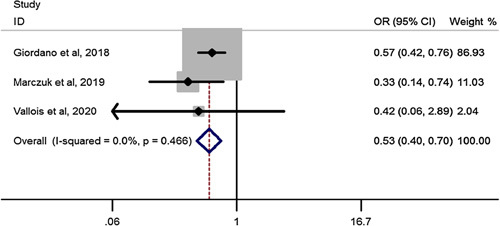
Forest plot to show hypertension remission after Roux-en-Y gastric bypass in elderly compared to youngers than 60 years. OR, odds ratio.

Pooled estimation of a meta-analysis of odds ratio studies reported an OR of 0.53, that is in patients undergoing RYGB bariatric surgery, the chance of HTN remission decrease by 47% (OR: .53, CI 95%: 0.40–0.70) in elderly compared to youngers than 60 years(Fig. 10).

### Mortality after RYGB in elderly compared to youngers than 60 years

**Figure 11:**
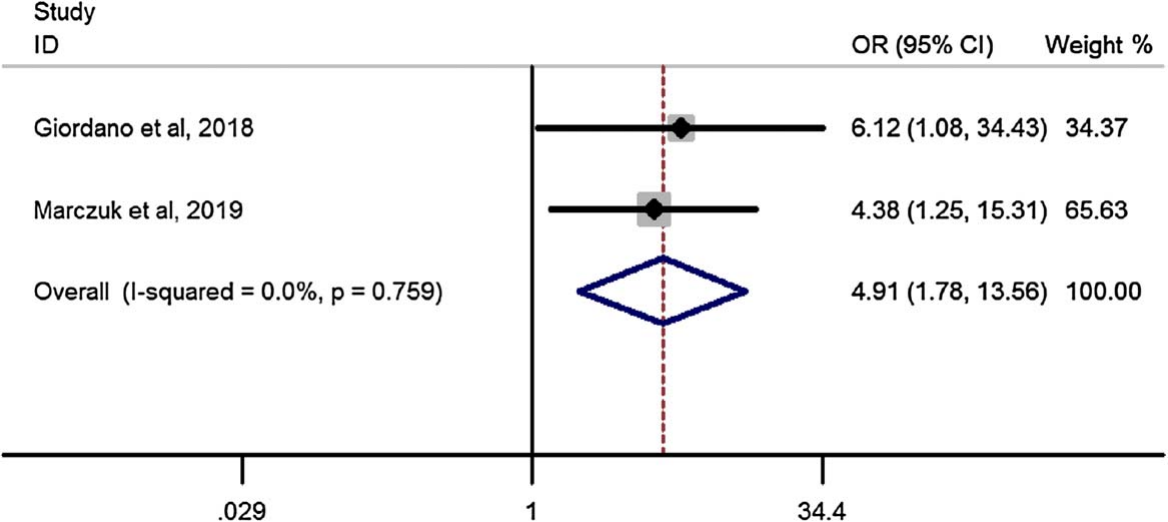
Forest plot to show mortality after Roux-en-Y gastric bypass in elderly compared to youngers than 60 years. OR, odds ratio.

Pooled estimation of a meta-analysis of odds ratio studies reported an OR of 4.91, that is in patients undergoing RYGB, the chance of mortality increases by 4.91 times (OR: 4.91, CI 95%: 1.78–13.56) in elderly compared to youngers than 60 years (Fig. 11).

### T2DM remission after RYGB in elderly compared to youngers than 60 years

**Figure 12:**
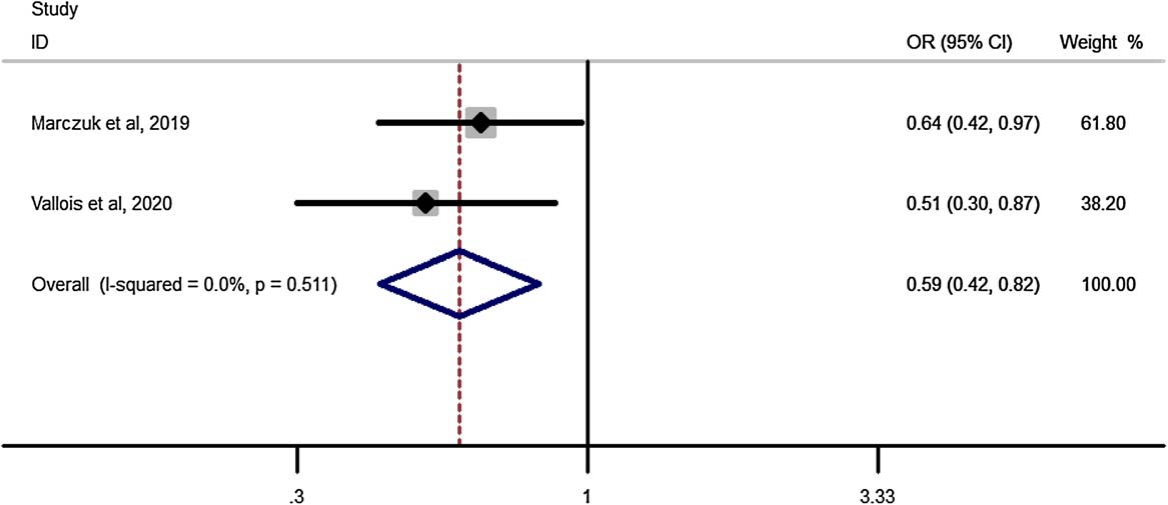
Forest plot to show type-2 diabetes mellitus remission after Roux-en-Y gastric bypass in elderly compared to Youngers than 60 years. OR, odds ratio.

Pooled estimation of a meta-analysis of odds ratio studies reported an OR of 0.59, that is in patients undergoing RYGB, the chance of T2DM remission decreases by 41% (OR: .59, CI 95%: 0.42–0.82) in elderly compared to youngers than 60 years(Fig. 12).

### Total complications after SG in elderly compared to youngers than 60 years

**Figure 13:**
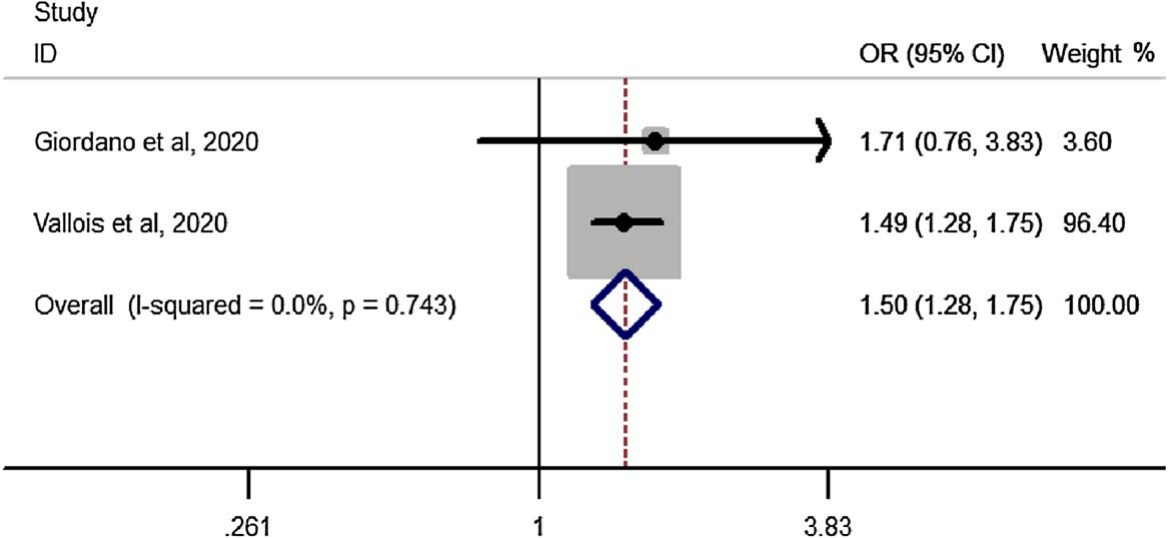
Forest plot to show total complications after sleeve gastrectomy in elderly compared to youngers than 60 years. OR, odds ratio.

Pooled estimation of a meta-analysis of odds ratio studies reported an OR of 1.5, that is in patients undergoing SG, the chance of total complication**s** increase by 50% (OR: 1.50, CI 95%: 1.28–1.75) in elderly compared to youngers than 60 years (Fig. 13).

### OSA remission after SG in elderly compared to youngers than 60 years

**Figure 14:**
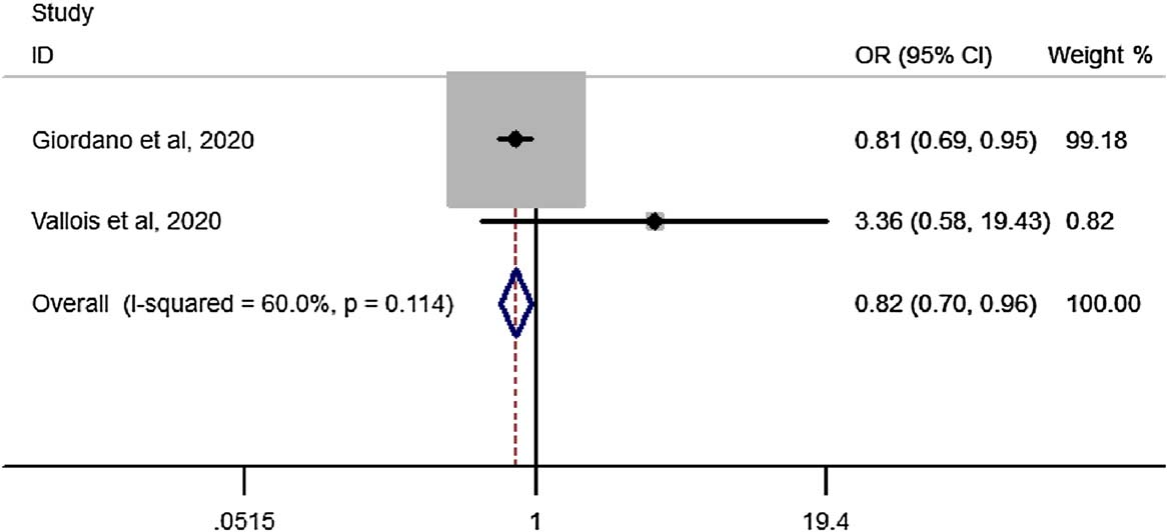
Forest plot to show OSA remission after sleeve gastrectomy in elderly compared to youngers than 60 years. OR, odds ratio; OSA, Obstructive sleep apnea.

Pooled estimation of a meta-analysis of odds ratio studies reported an OR of .82, that is in patients undergoing SG, the chance of OSA remission decreases by 18% (OR: .82, CI 95%: 0.70–0.96) in elderly compared to youngers than 60 years (Fig. 14).

### %EWL following RYGB in youngers than 60 years compared to elderly^23^^,^^25^^,^^35^,^36^,^37^,^38^,^39^

**Figure 15:**
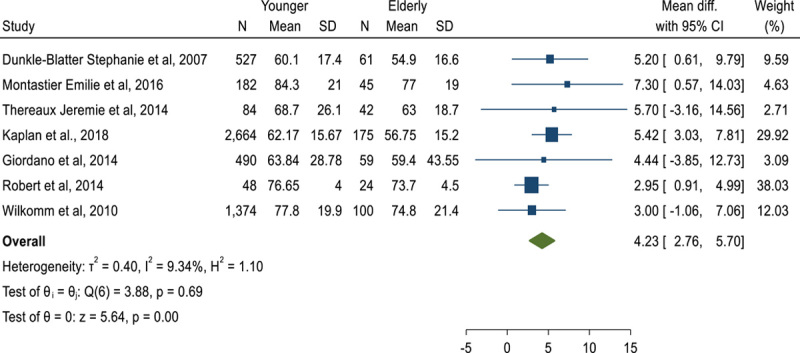
Forest plot to show %excess weight loss mean difference following Roux-en-Y gastric bypass in youngers than 60 years compared to elderly.

MD of %EWL after RYGB showed that youngers experience an extra 4.23%EWL compared to elderly people following RYGB (MD: 4.23, CI 95%: 2.76–5.70) (Fig. 15).

### %EWL following SG in youngers than 60 years compared to elderly^8^^,^^39^,^40^,^41^,^42^,^43^,^44^,^45^,^46^,^47^,^48^

**Figure 16:**
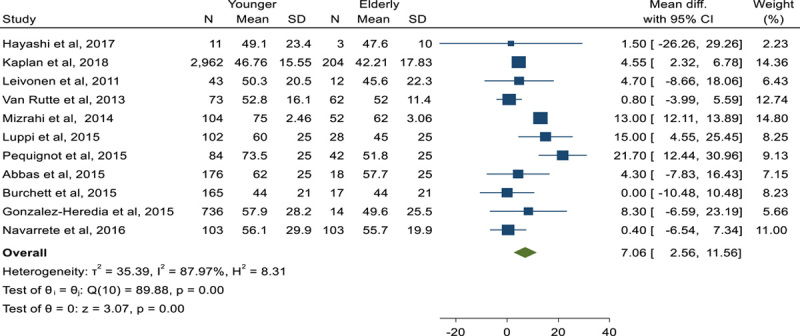
Forest plot to show %excess weight loss mean difference following sleeve gastrectomy in youngers than 60 years compared to elderly.

MD of %EWL following SG showed that youngers experience an extra 7.06%EWL compared to elderly people following SG (MD: 7.06, CI 95%: 2.56–11.56) (Fig. 16).

### %EWL following SG vs. RYGB in elderly^10^^,^^39^^,^^49^,^50^,^51^,^52^

**Figure 17:**
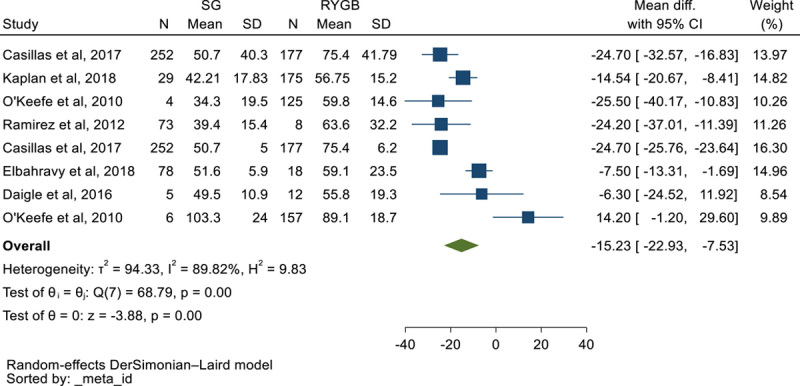
Forest plot to show %excess weight loss mean difference in elderly following sleeve gastrectomy vs. Roux-en-Y gastric bypass.

**Figure 18:**
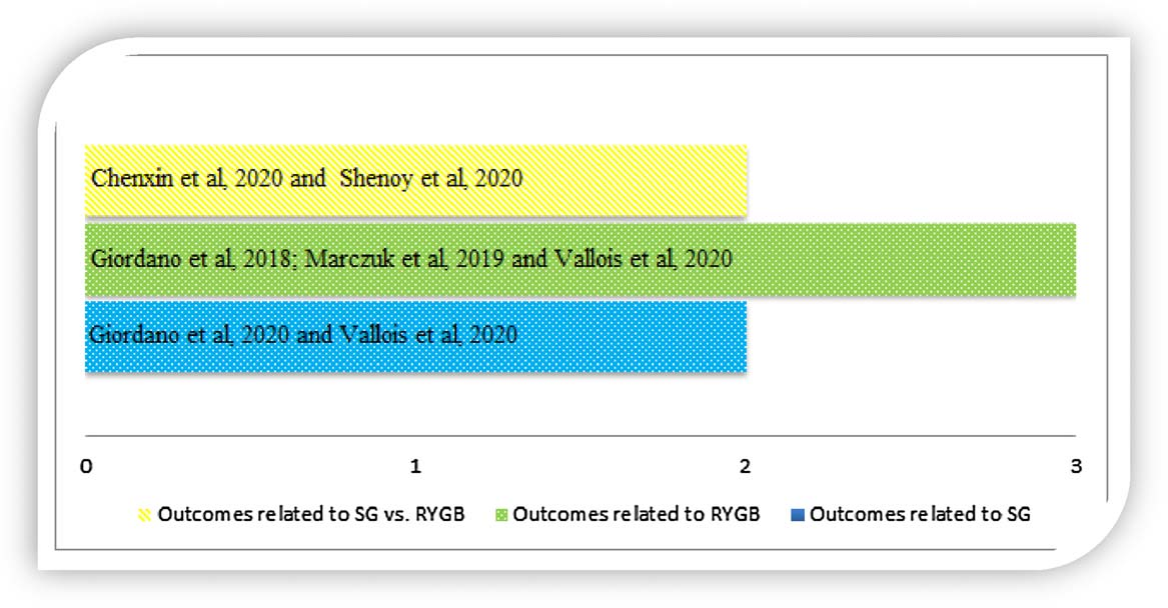
List of meta-analyses reporting outcomes related to only sleeve gastrectomy (SG), only Roux-en-Y gastric bypass (RYGB) and comparing SG vs. RYGB.

MD of %EWL following SG vs. RYGB, showed that the patients experience an extra 15.23%EWL following RYGB compared to SG (MD: −15.23, CI 95%: −22.93, −7.53), in other word SG leads to 15.23%EWL less than RYGB (Fig. 17) (Figure 18).

## Discussion

In the last decades life expectancy has significantly increased in the most developed countries, where the rates of obesity are also constantly growing^53,54^. Moreover, the diffusion of minimally invasive procedures and the improvement of perioperative anaesthetic management allowed to perform bariatric interventions in older patients.

For these reasons, there is currently a consistent body of literature proving that BMS can be safely and effectively performed in patients older than 60 years^55^. Some evidences seem to suggest that BMS has comparable results in younger and older patients^56^, while other studies have proved longer hospital stay and lower weight loss among patients older than 60 years^57^. Moreover, no consensus has been reached regarding the best procedure in terms of risks and outcomes.

SG is the most commonly performed procedure worldwide^58^, and a recent article has demonstrated that in older patients is as effective as in those younger than 60 years in terms of weight loss and improvement of comorbidities up to 5 years of follow-up^59^. Also, the meta-analysis of Giordano *et al*.^32^ has underlined the comparable results in terms of safety between older and younger patients, even if lower weight loss was recorded for the elderly ones.

On the other hand, a recent retrospective multi-institutional study from France showed that 90-day complication rate maybe higher for older patients undergoing RYGB when compared to younger ones^3^. Other articles did not find that older age was related to higher risks after RYGB but lower weight loss was demonstrated^3^.

A prospective trial^3^ has also evaluate 1-year outcomes of SG and RYGB in patients 65 years or older showing better weight loss after the RYGB. Currently, only one paper is available on one-anastomosis gastric bypass (OAGB) in this class of patients showing that the one anastomosis bypass can be a good choice because of its shorter operative time, higher efficacy and low complication rate^60^.

Serious morbidity following bariatric surgery is uncommon. Since laparoscopic sleeve gastrectomy (LSG) is associated with less adverse 30-day outcomes in comparison to RYGB^59^ in younger patients similar outcomes could be expected in the elder population. Indeed, our analysis showed that SG significantly decreases the risk of early and late complications and mortality in patients older than 60 when compared to RYGB.

However, despite the vast body of literature on BMS in elder patients, there are conflicting reports on complications. While some studies have demonstrated that patients younger than 65 and older than or equal to 65 years had similar perioperative morbidity and mortality after bariatric surgery^61^, other articles^17^ have reported that elderly patients have higher 30-day odds of serious complications and 30-day mortality. The present umbrella study further demonstrates that SG and RYGB both have higher rates of complications and mortality in patients with older than 65 years. In light of these increased risks, it is noteworthy that SG decreases the percentage of late complications and death when compared to RYGB suggesting that restrictive surgery maybe more appropriate in this class of individuals.

Moreover, little evidence has been published in septuagenarians confirming slightly higher rates of postoperative complications compared with a younger population^62,63^. Considering these outcomes, updated guidelines on BMS^64^ concluded that there is no upper patient-age limit to BMS and those older individuals who could benefit from BMS should be considered for surgery after careful assessment of comorbidities and frailty.

In terms of metabolic outcomes, our umbrella research confirmed that both remissions from diabetes and weight loss were effective in patients older than 65 years, but younger patients had better results.

Longer duration of diabetes^60^ causes an irreversible loss of beta-cells. Thus, the metabolic mechanism of the RYGB is predictably less effective in the older age when compared to younger patients. Interestingly, only HTN was significantly improved after RYGB when compared to LSG in our study, while no different effect was noted on OSAS and TD2M.

Several studies^17,61^ have clearly demonstrated that the RYGB provides better short and long-term weight loss than the LSG and this finding was also confirmed for older patients by our umbrella analysis.

Weight loss and maintenance is undoubtedly related to a regular physical activity after BMS^62,63^ and older individuals tend to experience early fatigue and to exercise less regularly than younger patients. However, all the available evidence and our meta-analysis show that BMS is still effective even if it induces lower %EWL results in the elderly population when compared to subjects younger than 60 years.

Although this evidence needs to be confirmed by prospective trials and have been partially previously reported by previous single studies and meta-analysis, to the best of our knowledge, we performed the first umbrella meta-analysis on this topic. The umbrella construction allows drawing an overall conclusion over conflicting results of previous meta-analyses.

The updated findings of this study provide insights into the current state of the literature, based on a total of 75 elderly patients included. A major constraint of this study is that the weight loss outcomes and remission of comorbidities may have been influenced by the varying operative techniques used for both RYGB and SG. For instance, research has demonstrated that creation a longer length of the biliopancreatic loop during RYGB can result in greater weight loss and a higher likelihood of remission from obesity-related medical problems^65^. Another limitation was that only three out of the six included meta-analyses had investigated the effect of both LSG and RYGB on elderly patients. Moreover, not all the outcome measures were studied in the included papers, therefore all the forest plots include a number of studies less than or equal to 3, and 10 of them include only 2 articles. Subsequently, despite the overall large sample size provided by the “umbrella meta-analysis”, the results of this paper mainly rely on the outcomes of 3 previous meta-analyses.

## Conclusion

This umbrella meta-analysis aims to settle different results of previous single meta-analysis on MSB in patients older than 60 years. Our outcomes suggest that BMS provides satisfactory outcomes in patients older than 60 years but with higher risks of complications. In the elderly population, SG is a safer surgical option than RYGB, which on the contrary induces better weight loss and remission of HTN.

## Ethical approval

No applicable (review of the literature).

## Source of funding

None.

## Author contribution

M.K.: conceived the idea for the topic, data gathering, consulting and writing. M.M.: consulting and reviewing. R.V.: statistics and methodology. S.S.S.: data gathering, consulting and reviewing. A.V.: data gathering, consulting and reviewing. M.M. and R.V.: organization leadership, data gathering, consulting and writing.

## Conflicts of interest disclosure

None.

## Research registration unique identifying number (UIN)

1. Name of the registry: Prospective Register of Systematic Reviews (PROSPERO).
2. Unique Identifying number or registration ID: *CRD42022318906.*
3. Hyperlink to your specific registration (must be publicly accessible and will be checked): CRD42022318906 (Available at https://www.crd.york.ac.uk/prospero/display_record.php?RecordID=318906) Dear colleague: The above mentioned link can be checked, it is correct but maybe due to the policy of the PROSPERO it cannot be hyperlinked, please check it, maybe you can slove it, thanks a lot.

## Guarantor

Rohollah Valizadeh and Mario Musella.

## Provenance and peer review

Not commissioned, externally peer-reviewed.

## Data availability statement

All authors declare that data of this meta-analysis were retrieved from published literature where they are available.
